# Hidden gout- Ultrasound findings in patients with musculo-skeletal problems and hyperuricemia

**DOI:** 10.1186/2193-1801-3-592

**Published:** 2014-10-09

**Authors:** Monika A Reuss-Borst, Cornelia A Pape, Anne K Tausche

**Affiliations:** Rehabilitation Clinic for Rheumatology and Oncology, Kurhausstr 9, 97688 Bad Kissingen, Germany; Department of Rheumatology, University Clinic “Carl-Gustav-Carus”, Dresden, Germany

**Keywords:** Gout, Hyperuricemia, Ultrasonography, Pain

## Abstract

The goal of this study was to investigate the frequency of gout-specific ultrasonography findings in a cohort of hyperuricemic patients with various musculo-skeletal complaints.

A blinded examiner regarding the patients’ clinical and laboratory assessment performed standardized ultrasound examinations of 12 joints in 74 individuals with diverse musculo-skeletal complaints. Gout-specific changes were assessed and combined with the patients’ medical history (diagnosis gout vs. non-gout) and laboratory values of hyperuricemia.

Of 74 patients, 58 (mean age 55 yrs) had hyperuricemia (serum uric acid levels > 7 mg/dl/420 μmol/L). Of those, 27 (47%) had a history of gout attacks.

In total, 888 joints were examined by ultrasound. With 44/324 joints (14%) the pathological finding most often found in joints of gout patients was the double contour sign compared to 29/372 joints (8%) in patients with asymptomatic hyperuricemia and 2/192 joints (1%) in normouricemic controls.

In patients with gout, the ultrasound showed pathological findings in 67/324 joints (21%). In 26/39 (67%) previously affected joints, gout-specific sonographic indications were found. With regard to the first metatarsophalangeal joint, sonographic pathologies were detectable in 16/22 (73%) so far asymptomatic joints on the contralateral.

Ultrasonographic gout-specific signs are not only found in joints affected by gout attacks, but often also in the corresponding contralateral, asymptomatic joint. Patients with asymptomatic hyperuricemia already showed sonographic features implicating an as yet “silent” precipitation of urate crystals. As the examined cohort represents patients at high risk, further research for gout-specific findings is indicated, especially for hyperuricemic patients.

**Electronic supplementary material:**

The online version of this article (doi:10.1186/2193-1801-3-592) contains supplementary material, which is available to authorized users.

## Introduction

Gout is one of the most common forms of inflammatory arthritis in men with increasing prevalence. It is typically characterized by two clinical phases. Normally, the initial phase of the disease is marked by acute attacks of active synovitis, which spontaneously resolves with asymptomatic periods between flares (intercritical gout). Without adequate treatment of hyperuricemia (HU) as the underlying condition, it may transit into a chronic (tophaceous) disease with polyarticular manifestations and crystal deposition in soft tissues, so called tophi (Neogi 2011). Although Neogi et al. found that up to 2/3 of patients with at least one gout attack in the previous year had recurrent attacks (Neogi et al. 2006), in daily practice, patients often present the “intercritical phase” with unspecific musculo-skeletal complaints mainly associated with other concomitant diseases such as diabetes mellitus or arterial hypertension to their general practitioners/doctors.

In this early phase of disease, it may be difficult or even impossible to confirm the diagnosis of gout, in particular since the definitive confirmation of gout rests on the identification of monosodium urate (MSU) crystals in the synovial fluid (Zhang et al. 2006).

In the last decade, the role of musculoskeletal ultrasound (US) in investigating MSU deposition is rapidly evolving since various findings have been described as specific for gout such as the hyperechoic enhancement of the superficial margin of the hyaline cartilage (double contour sign) (Thiele & Schlesinger 2007; Chowalloor & Keen 2013). Thus these new imaging techniques might assist in the diagnosis of gout, in particular in confirming early structural damage. However, little is known about whether gout-specific signs are only identified in clinically involved joints, or also in as yet (clinically) unaffected joints. In addition, we do not know whether the “silent” MSU deposition might be responsible for those unspecific symptoms in patients with HU and unspecific musculo-skeletal problems. Furthermore, there is still no answer to the question if ultrasonographic signs of MSU precipitation are predictable in patients with HU for the development of clinically manifest gout.

Since HU is the most important risk factor for gouty arthritis and a common finding in men (21,1%) and women (4,7%) with often concomitant unspecific joint complaints (Zhu et al. 2011), US might be a valuable tool to identify hyperuricemic patients at risk for gouty arthritis.

In order to investigate the usefulness of US in patients with HU and (unspecific) various joint complaints we performed a prospective blinded study using patients with HU as well as normouricemic controls.

## Material and methods

### Patients

74 adult patients in an inpatient rehabilitation program for musculo-skeletal problems were included in the study. All patients were considered to be at high risk for occupational disability and therefore underwent an inpatient rehabilitation program for the statutory pension fund because of their various joint and bone complaints. Demographic, clinical and laboratory characteristics such as uric acid, blood glucose level, cholesterol, triglycerides and GGT of each patient were recorded. All patients filled in a questionnaire about the history of previous gout attacks, risk factors for gout and individual knowledge about nutritional recommendations. A detailed clinical examination with special emphasis on joint pathology and presence of clinical tophi was performed in all patients by an expert clinical rheumatologist recording swelling and tenderness of joints.

Prior to inclusion, all patients provided informed consent for participation. The study was in accordance with the declaration of Helsinki and approved by the ethical committee of the State Chamber of Physicians of Bavaria.

### Ultrasonographic examinations

US examinations were performed independently by a second (trained, DEGUM certified) rheumatologist who was blinded to the clinical and laboratory data of the patients. All US examinations were performed using a MyLab 70 XVG (Esaote SpA, Genoa, Italy) equipped with 2 multi-frequency linear probes operating at a frequency spectrum from 7 to 15 MHz.

Standardized examinations were carried out in 12 joints (6 joints bilaterally) for each patient: first metatarsophalangeal joints (MTP I), upper ankle (talonaviculare) joints, knee joints, wrists, thumb basal joints (MCP I) and elbows. All studies were performed in two dimensions by scanning across the joints and moving the probe from medial to lateral and from distal to proximal. Each site was scanned in gray scale mode to detect structural changes and Power Doppler technique to detect abnormal blood flow.

Beside detection of synovitis and erosions, gout-related lesions were investigated in each joint, including the presence of the double contour sign (DC) and peri-/intraarticular hyperechoic cloudy areas (HC).

DC was defined as focal or diffuse echogenic enhancement on the superficial margins of the joint cartilage. HC was characterized as peri-/intraarticular heterogeneous masses composed of hyper- or hypoechoic material sometimes possessing posterior shadows. Synovialitis was defined as the presence of either synovial fluid and/or synovial hypertrophy, which is characterized by abnormal hypoechoic intraarticular tissue that is poorly compressible and may exhibit a Doppler signal (show microvascular blood flow). Erosions were defined as intra-articular discontinuity of the bone cortex that were visible in at least two perpendicular planes (Thiele & Schlesinger 2007; Grassi et al. 2006; Perez-Ruiz et al. 2009). Sonographic pictures of each location in every patient were stored.

The clinical assessor combined clinical and sonographic data obtained in the study. A descriptive analysis of the data was performed. Demographic and clinical features were summarized as mean values for continuous variables and absolute frequencies or proportions (percentages) for categorical variables. Pairwise comparisons between frequencies of clinical data in different groups of patients were analyzed using the chi-square-test.

## Results

### Demographic and clinical characteristics

74 patients at the mean age of 54 years were studied. The most frequent main diagnosis on admission was back pain (61%) classified as M45 (35%), M53 (18%) or M51 (8%) according to the ICD-10-GM (International Classification of Disease). In 11/74 patients (15%) shoulder problems were mentioned as main diagnosis, 18/74 (24%) had other musculo-skeletal problems coded as M-diagnosis according to the ICD.

Gout as the underlying disease (M10) was mentioned in 0/27 (0%) patients with a history of gout by the admitting physician as main diagnosis. As first concomitant diagnosis it was mentioned in 3/27 (11%) cases, as second concomitant diagnosis in 8/27 (30%) and third concomitant diagnosis 4/27 (15%) cases. The clinical and laboratory characteristics of the study population are shown in Table 1.

**Figure 1:**
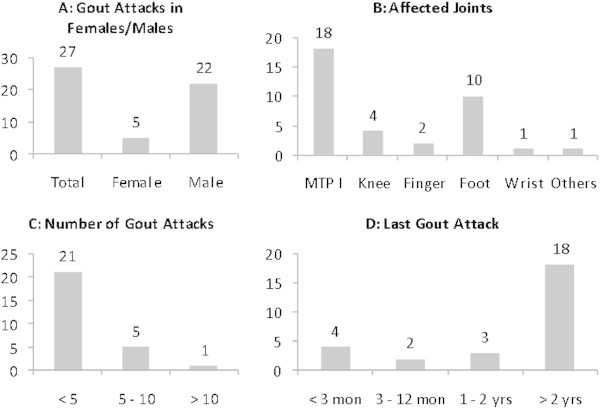
**Characteristics of gout attacks in our study population. A)** In our population more men than women were affected by gout. **B)** MTP I and foot were the joints most often affected. **C)** Most patients reported having had less than 5 gout attacks so far **D)** Most reported gout attacks had ocurred more than 2 years before the study.

Of 58 hyperuricemic patients (including those with a history of gout), 40 patients had been told by their primary physician that serum uric acid (SUA) had been elevated in the past. Twenty (35%) had received nutritional advice, 38 (65%) had never got information about life-style modification e.g. restriction of purine-rich food. Nevertheless, 41/58 (71%) patients knew that excessive drinking of beer and eating of grilled meat might trigger a gout attack, but only 7/58 (12%) knew about the urate-elevating effect of fructose-rich drinks. 27/58 (47%) investigated subjects had a previous history of gout attacks. Details of the involved joints and frequency of attacks are depicted in Figure 1. Of the 27 patients with gout 6 (22%) underwent urate lowering treatment during the study period, 10 (37%) had undergone it in the past and 11 (41%) had never been treated.

**Table 1:** **Clinical characteristics of the study population**

Clinical and laboratory characteristics	Gout (n = 27)	Asymptomatic hyperuricemia (n = 31)	Normouricemic controls (n = 16)
Mean Age, yrs	54,2	54,7	52,5
Male,%	81,5	61,3	56,2
Mean serum uric acid value in mg/dl (mmol/l))	7,7 (458,1)	8,1 (481,9)	5,2 (309,4)
BMI (kg/m^2^)	30,0	31,5	28,6
Triglycerides in mg/dl (mmol/l))	199,0 (2,269)	201,3 (2,295)	128,6 (1,466)
Cholesterol in mg/dl (mmol/l)	215,9 (5,613)	232,7 (6,05)	224,1 (5,827)
Glucose in mg/dl (mmol/l)	101,3 (5,622)	105,9 (5,877)	94,4 (5,239)
GGT (U/l)	103,0	61,9	38,9

16 patients with similar musculo-skeletal symptoms, but with SUA levels < 7 mg/dl were also included in this study.

### Ultrasonographic results in HU and normouricemic controls

**Figure 2:**
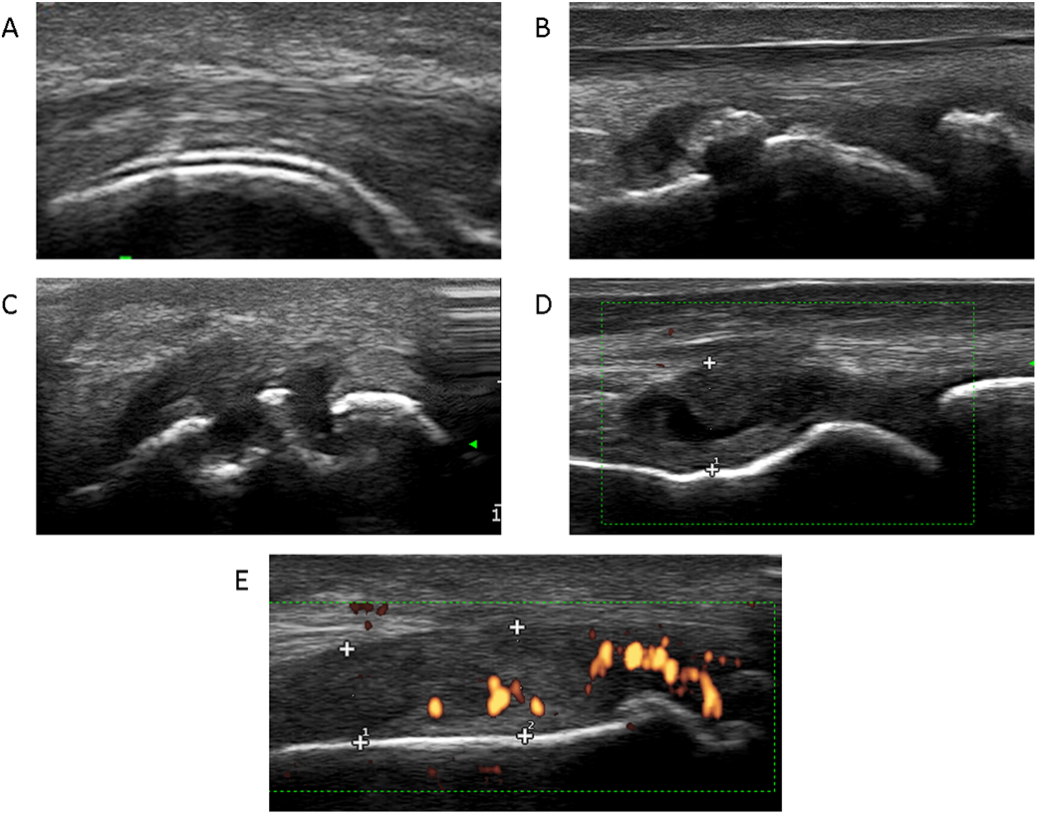
**US findings of MSU deposits in joints. A)** the typical hyperechoic enhancement on the cartilage surface (“double contour sign”) **B)** intra-articular hyperechoic clouds **C)** bone erosion and **D)** active synovitis with **E)** a periarticular power signal.

The results of the ultrasonographic examinations are shown in detail in Figure 2 and Additional file 1: Table S1, Tables 2 and 3. In total, 888 joints in 74 patients were investigated. The DC sign was the pathological finding described most often, in particular in 44/324 joints (14%) of gout patients, 29/372 (8%) joints of HU patients and 2/192 (1%) normouricemic controls. Thus, DC signs were significantly more often described in joints of gout patients than in joints of HU patients (*χ*^2^(1) = 6,172, p = .026) and normouricemic controls (*χ*^2^(1) = 23,342, p < .001). HU patients also showed significantly more DC signs than normouricemic controls (*χ*^2^(1) = 11,121, p = .001).

**Table 2:** **Sonographic findings of 31 patients with (asymptomatic) hyperuricemia**

Pat.-Nr.	MTP I R	MTP I L	Ankle R	Ankle L	Knee R	Knee L	Wrist R	Wrist L	TBG R	TBG L	EB R	EB L
**01**	HC, S		DC									
**02**												
**03**	HC	HC					HC	HC	HC			
**04**	DC, HC	DC, HC, S		DC								
**05**												
**06**	DC	DC, HC	DC	DC								
**07**		E										
**08**	S	S										
**09**		DC, HC									DC	DC
**10**		DC									DC	DC
**11**	HC, S	DC										
**12**												
**13**												
**14**												
**15**												
**16**	DC, HC, S											
**17**	S	S, E										
**18**												
**19**	DC, HC, S, E	HC						E	HC			
**20**	DC, S, E											
**21**	DC, HC, S, E	DC, HC, S										
**22**									HC			
**23**	DC	DC										
**24**	HC, S, E	HC, E										
**25**	DC	DC										
**26**												
**27**												
**28**		HC, S		DC	DC	DC						
**29**												
**30**	DC, HC, E					DC, HC, S						
**31**		HC, S										

*Abbreviations*: *DC* double contour sign, *HC* hyperechoic clouds, *E* erosions, *S* synovitis.

**Table 3:** **Sonographic findings of 16 patients with normal serum urate values**

Pat.-Nr.	MTPI R	MTPI L	Ankle R	Ankle L	Knee R	Knee L	Wrist R	Wrist L	TBG R	TBG L	EB R	EB L
**01**												
**02**												
**03**												
**04**												
**05**												
**06**	HC											
**07**												
**08**												
**09**												
**10**												
**11**			HC	DC								
**12**	DC	HC							E			
**13**												
**14**							S, E	S,E				
**15**		S, E										
**16**												

*Abbreviations*: *DC* double contour sign, *HC* hyperechoic clouds, *E* erosions, *S* synovitis.

In patients with gout, pathological ultrasonographic findings were detectable in 67/324 joints (21%). When correlated with the clinically affected localization, in 26/39 (67%) gout-specific sonographic findings were found by the blinded investigator. With regard to the MTP I, sonographic pathologies were detectable in 16/22 (73%) MTP I joints on the contralateral, till now asymptomatic side. In only 3/26 (12%) previously affected MTP I joints, the sonographic findings were judged to be normal. In 4/27 (15%) patients DC signs were found in up to the present unaffected knees, all four patients having a history of previous podagra (gout attacks in MTP I joints). In 5 of the 27 gout patients, hyperechoic clouds were also described in so far unaffected wrists (Additional file 1: Table S1).

In patients with HU without history of typical gout, 52 of 372 investigated joints (14%) showed pathological findings with US. In only 9/31 patients (29%) with HU, the sonography in the investigated joints panel was judged to be completely normal. The DC sign was found in 17/62 (27%) investigated MTP I joints. In 17/31 patients (55%) the investigator described echogenic structures and/or double contour signs in MTP I joints; in 5/31 patients a synovialitis and erosions were found in the MTP I joints (Table 2).

### Normouricemic controls

In 11/192 investigated joints (6%) pathological findings without clinical correlates were reported (Table 3).

## Discussion

The majority of our patients with musculo-skeletal problems and HU had previously been informed about elevated serum urate levels by their primary care physicians. Beer and grilled meat are known to be risk factors for high serum urate levels by most patients. However, less than half of these patients had received certain diet and lifestyle recommendations in the past for lowering serum urate levels, which is in accordance with previous studies (Doherty et al. 2012). Similarly, only a minority (12%) knew about the urate-elevating effects of soda (fructose sweetened) drinks. This finding underlines the necessity for more information on diet and lifestyle measures even if clinical trials on non-pharmacologic interventions have only reported a 10-18% decrease in serum urate (Khanna et al. 2012).

In our study, we found a surprisingly high percentage of patients with a gout history among the hyperuricemic population, which might at least be partially explained by the selected study population of patients with longstanding numerous musculo-skeletal problems. Those patients were admitted to our rehabilitation unit by their primary care physician because of their high risk for occupational disability. In addition, our study population exclusively comprised manual workers (“blue collar” jobs) with mostly low socioeconomic status. Although gout was mentioned as concomitant diagnosis in >50% of the patients with a history of gout attacks, gout was never mentioned as main diagnosis in our study population. In 12 of 27 (44%) patients with a history of gout, this diagnosis was not at all documented by the admitting physician. Thus, gout is probably still considered to be a benign disease by many doctors, not worth being mentioned in the admitting letter. Correspondingly, only 6 of 27 (22%) gout patients currently received a urate-lowering treatment with none of the medically treated patients reaching the target level below 6 mg/dl as recommended by current guidelines (Khanna et al. 2012).

Hence, one first important result of our study is that gout is quite frequent, but underreported in the diagnosis in the admission letter and furthermore remains undertreated or even untreated, particularly in this study population with longstanding musculoskeletal problems and with a high risk of occupational disability.

Claessen et al. ( 2010) have similarly investigated the role of SUA as a predictor of occupational disability. Musculoskeletal disorders cause the largest proportion of all cases of disability pension among manual workers. An age-adjusted analysis revealed a strong positive association between elevated SUA concentration and occupational disability due to musculo-skeletal disorders in a cohort of 16532 male construction workers in Germany who underwent occupational health examinations from 1986 to 1992 and were followed until 2005.

In accordance with this, our study population comprised patients with a longstanding history of musculo-skeletal complaints. It is known that crystallization of MSU is facilitated in osteoarthritic joints, which might explain the high prevalence of pathological findings in our selected population.

One might further speculate that the unspecific musculoskeletal problems in our study population might at least partially by due to subclinical MSU crystal deposition for example in the small joints of the spine. This may be in accordance with a recent study of Konatalapalli et al. ( 2012) that reported on axial gout as a common feature of chronic gouty arthritis.

With regard to the sonographic examinations performed by a blinded rheumatologist we found a high prevalence of gout-specific lesions in so far clinically involved, but –more interestingly also – in so far clinically uninvolved joints. This applies in particular for the MTP I joints as gout index joints. The presence of MSU crystals in the synovial fluid from asymptomatic individuals with HU has already been demonstrated by polarized light microscopy more than 30 years ago (Rouault et al. 1982). Thus, it is not surprising that with better imaging techniques, gout specific structural lesions become visible. Serum urate seems to have a preference to crystallize on the surface of (damaged) cartilage with the normal components of cartilage chondroitin sulfate and phosphatidylcholin facilitating nucleation and crystallization of MSU explaining the high prevalence of the DC signs in patients with gout, but also HU. In particular, the DC sign has not been described in other disease entities until now and must be interpreted as highly specific for gout with a specificity >98% (Ottaviani et al. 2012). Our results are in accordance with a previous study of Pineda et al. (Pineda et al. 2011) who found the DC sign in 25% of the first MTP joints from hyperuricemic individuals.

The high prevalence of pathological US findings in our study population might support the hypothesis that US can help to identify patients with HU who have a high risk for developing clinically manifest gout. With these patients, it might be worthwhile initiating long-term urate-lowering therapy early in the course of disease, in particular since we know that uric acid is a danger signal of increasing risk for osteoarthritis through NLRP inflammasome activation leading to the production of IL-18 and IL-1ß. Recently, Denoble et al. (Denoble et al. 2011) could confirm the potential involvement of the innate immune system in osteoarthritis (OA) pathology and OA progression by correlating synovial fluid uric acid with IL-1ß and IL-1 and with OA severity.

Our study has several strengths and weaknesses. First of all, this is to our knowledge the first report on such a selected (high-risk) patient group with longstanding mainly unspecific musculo-skeletal problems and HU. The combination of clinical data and sonographical findings may give some new idea about possible underlying mechanisms of the patients’ complaints. Gout diagnosis was made clinically from the patient’s charts (physician-diagnosed) or history (self-reported) and was not confirmed by synovial fluid analysis which might be considered to be a weakness of this study. On the other hand, all patients did not have gout attacks during their hospital stay, so we considered an invasive procedure such as synovial fluid aspiration not to be appropriate. US was performed by an independent blinded observer and was the only imaging technique used to detect joint pathologies. Ultrasonographic findings were compared with the clinical examinations of the joints. Other imaging techniques as comparator were not used.

The US was done in a standardized manner using a 12 joint (6 joints bilateral including the MTP I) examination protocol modified from investigations in patients with rheumatoid arthritis. To date, no standardized protocol for gout patients exists.

In conclusion, our study contributes some evidence that longstanding, untreated HU might cause joint damage, even if asymptomatic, probably presenting as diverse musculo-skeletal complaints. Thus – if ultrasonographic gout-specific changes are detectable – “asymptomatic hyperuricemia” might be better termed as “subclinical or hidden gout”. US seems to be a suitable tool to early detect these structural changes. Our observations might have impact on further treatment decisions, in particular initiating urate-lowering therapy earlier, especially in patients with a high risk of occupational disability due to musculo-skeletal conditions. Further interventional trials are necessary to investigate if urate-lowering therapy has positive effects on those patients with HU and various musculo-skeletal problems.

## Electronic supplementary material

Additional file 1: Table S1: Sonographic findings of 27 patients with a history of gout. Blue fields: patients with tophi on clinical examination; Grey fields: joints with previous gout attacks, Abbreviations: DC=double contour sign, HC=hyperechoic clouds, E=erosions, S=synovitis. (DOCX 20 KB)
