# Supplementary material for: Hidden gout- Ultrasound findings in patients with musculo-skeletal problems and hyperuricemia
Source: Springerplus. 2014 Oct 9;3:592. doi: 10.1186/2193-1801-3-592 (PMC4203789; doi:10.1186/2193-1801-3-592)
Supplement: Supplementary file 1 — Additional file 1: Table S1: Sonographic findings of 27 patients with a history of gout. Blue fields: patients with tophi on clinical examination; Grey fields: joints with previous gout attacks, Abbreviations: DC=double contour sign, HC=hyperechoic clouds, E=erosions, S=synovitis. (DOCX 20 KB) [file 40064_2014_1303_MOESM1_ESM.docx]

| **Pat.-Nr.** | **MTP I R** | **MTP I L** | **Ankle R** | **Ankle L** | **Knee R** | **Knee L** | **Wrist R** | **Wrist L** | **TBJ R** | **TBJ L** | **EB R** | **EB L** |
| --- | --- | --- | --- | --- | --- | --- | --- | --- | --- | --- | --- | --- |
| **01** | DC, HC, E | DC, HC, S |  |  |  |  |  |  |  |  |  |  |
| **02** | S |  | E |  |  |  | S | S |  |  |  |  |
| **03** |  | DC |  |  | DC |  | HC |  |  |  |  |  |
| **04** | HC |  |  |  | DC |  | HC |  |  |  |  |  |
| **05** | DC | DC, HC, S |  |  |  |  |  |  |  |  | DC | DC |
| **06** | HC, S |  |  |  |  |  |  |  |  |  |  |  |
| **07** |  |  |  |  |  |  |  |  |  |  |  |  |
| **08** | DC, HC, E | DC, E, S |  |  |  |  |  |  |  |  |  |  |
| **09** | DC, HC | DC, HC |  |  |  |  |  |  | HC |  |  |  |
| **10** |  |  |  |  |  |  |  |  |  |  |  |  |
| **11** |  |  |  |  |  |  |  |  |  |  |  |  |
| **12** | DC, HC. E | DC, HC |  |  | S | S |  |  |  |  |  |  |
| **13** | HC, S, E | DC, HC, E |  |  |  |  |  |  |  |  |  |  |
| **14** | DC, HC, S | DC, HC, S |  |  |  |  |  |  |  |  |  |  |
| **15** | E | DC, S |  |  |  |  |  |  |  |  |  |  |
| **16** | DC, HC, S, E | DC, E |  |  |  |  | HC | HC |  |  |  |  |
| **17** | DC, HC, S, E | DC, HC |  |  |  |  |  |  | E |  |  |  |
| **18** | DC, HC, S, E | DC, S |  |  |  |  |  |  |  |  |  |  |
| **19** |  |  |  | DC |  |  |  |  |  |  |  |  |
| **20** | DC, HC | DC, HC |  |  |  | DC |  |  |  |  |  |  |
| **21** |  | DC | DC |  | DC | DC |  |  |  |  |  |  |
| **22** | HC, E | DC, E |  |  |  |  |  |  |  |  |  |  |
| **23** | DC, HC, E | DC, HC, E |  |  | DC | DC |  |  |  |  |  |  |
| **24** | DC, HC | DC, HC |  |  |  |  |  |  |  |  |  |  |
| **25** |  | DC, HC, S |  |  |  |  |  |  |  |  |  |  |
| **26** | HC | DC, HC, S |  |  |  | HC, S | E | HC, E |  |  |  |  |
| **27** | DC, HC, S | DC, HC, E |  |  |  |  |  | HC, E | E |  |  |  |
